# Is the European badger a new host for *Dirofilaria immitis*? The first records in Greece

**DOI:** 10.1007/s00436-024-08141-0

**Published:** 2024-02-01

**Authors:** Grigorios Markakis, Georgios Sioutas, Dimitra Bitchava, Anastasia Komnenou, Maria Ganoti, Elias Papadopoulos

**Affiliations:** 1 Laboratory of Parasitology and Parasitic Diseases, School of Veterinary Medicine, Faculty of Health Sciences, Aristotle University of Thessaloniki, 54124 Thessaloniki, Greece; 2 Vet In Progress Plus, Veterinary Laboratories, Agia Paraskevi, 15343 Attiki, Greece; 3 Exotic and Wildlife Medicine Unit, Department of Clinical Studies, School of Veterinary Medicine, Faculty of Health Sciences, Aristotle University of Thessaloniki, 57400 Thessaloniki, Greece; 4 ANIMA – The Association for the Protection and Welfare of Wildlife, 17676 Kallithea, Greece

**Keywords:** *Dirofilaria immitis*, Cardiovascular dirofilariosis, European badger, Zoonotic potential, Wildlife reservoir hosts, Greece

## Abstract

*Dirofilaria immitis* is a ubiquitous nematode parasite with zoonotic potential, transmitted by mosquitoes, that causes heartworm disease in various animal species. Dogs are the parasite’s typical final host, and wild carnivores represent the parasite’s reservoir in nature. Studies on *D. immitis* infections in wild animals are essential to assess infection pressure for domestic animals, and until now, there has been only one infection case reported in a European badger (*Meles meles*). The current report describes the first two European badger cases with cardiovascular dirofilariosis in Greece. Two adult male badgers were rescued in Heraklion and Chania, Crete Island, and admitted to “ANIMA -Wildlife Rehabilitation Centre” in Athens. The detailed clinical examination revealed that the first badger suffered from severe broncho-pneumonitis while the second one displayed clinical signs associated with severe brain trauma. Blood samples were taken for haematology and biochemistry analyses during their short hospitalisation period. In addition, different routine diagnostic tests were carried out, including heartworm antigen testing (ELISA) and the modified Knott’s test for microfilariae. Both badgers were positive in both tests. The animals died a few hours after their admission and the detailed necropsies followed, revealed the presence of three parasites in each animal’s right heart, morphologically identified as adults of *D. immitis*. These findings add the European badger in the list of additional potential reservoir hosts for *D. immitis* and highlight the potential role of wildlife for companion animals and human health.

## Introduction

Heartworm infection or cardiovascular dirofilariosis, caused by the filarioid nematode *Dirofilaria immitis, *is a disease that severely affects the cardiovascular and respiratory systems of many mammalian carnivore species globally (Tolnai et al. 2014; Moroni et al. 2020). The parasite has an indirect life cycle involving several mosquito species that can serve as intermediate hosts, most belonging to the genera *Aedes* and *Culex* (Tolnai et al. 2014; Moroni et al. 2020; Ionică et al. 2022).

The adult worms are located in the host’s pulmonary arteries and in the right ventricle (Otranto and Deplazes 2019). Female adults release microfilariae (L1) in the host’s bloodstream, where Culicidae mosquitoes ingest them during their blood meal. Inside the mosquito, in approximately 2 weeks, the first stage larvae develop into L2 and then L3, the parasite’s infective stage for the final hosts. The mosquito infects the host with L3 during the bloodmeal. In the final hosts, the L3 develop into L4 inside the subcutaneous tissue before migrating through the adipose or skeletal muscle tissue and developing to L5 (immature adults). Finally, the L5 enter the host’s vascular system and migrate to the heart and pulmonary arteries (Hoch and Strickland 2008).

Apart from its veterinary importance, *D. immitis* is also of public health concern due to its zoonotic potential (Tolnai et al. 2014; Ionică et al. 2022). The infection is not patent in humans, but the larvae can cause lung lesions resembling neoplasms (Tolnai et al. 2014). Dirofilariosis cases are most frequently reported in southern European and Mediterranean countries. Greece, especially, is considered to be endemic for canine cardiac dirofilariosis, with its northern areas recording high prevalence of infection by *D. immitis* (Tolnai et al. 2014; Diakou et al. 2016). Although patent infections are most commonly described in domestic dogs (Ionică et al. 2022), the primary domestic reservoirs of *D. immitis*, the parasite has also been documented in a large variety of wild mammals, including red foxes (*Vulpes vulpes* Linnaeus, 1758), golden jackals (*Canis aureus* Linnaeus, 1758), European wildcats (*Felis silvestris silvestris* Schreber, 1777), and wolves (*Canis lupus* Linnaeus, 1758). Furthermore, cardiovascular dirofilariosis is considered one of the leading causes of death in wildlife animals (Wixsom et al. 1991). Most of these animal species thrive in Greece, but their role as definitive hosts for this parasite has not been adequately investigated. Currently, there is a lack of data regarding the epidemiological role of wildlife in transmitting *D. immitis* (Moroni et al. 2020).

Studies in wildlife reveal that the prevalence of *D. immitis* seems to be affected by many factors, such as climate change (global warming will probably support the altitudinal dissemination of the parasite) (Genchi et al. 2009; Simón et al. 2012; Otranto et al. 2013; Moroni et al. 2020), global movements (Tatem et al. 2006), and geographic locations (differences in the distribution of the vectors and in the temperatures which are favourable to the completion of the parasite’s life-cycle) (Gortazar et al. 1994; Moroni et al. 2020). Moreover, concerning the prevalence of *D. immitis*, gender, and BCS are not significant risk factors, while the infection risk increases with age, not because older animals are more susceptible than younger ones but because they have had higher exposure to the parasite (Gortazar et al. 1994; Rossi et al. 1996; Moroni et al. 2020).

“ANIMA-Association for Wildlife Care and Protection” is Greece’s biggest wildlife rehabilitation centre, treating more than 6000 wild animals annually. Every patient admitted in ANIMA’s first aid station in Athens, is recorded in the Entry Book along with all the accompanying information and history, as well as in AΝΙΜΑ’s electronic database. Each animal receives a code number describing its relevant information and the month of admission.

The first *D. immitis* infection in a European badger (*Meles meles* Linnaeus, 1758) was recorded in Romania in 2022, but it remained unclear if the badger was an accidental host. Despite its history of being a severely threatened species, the European badger is now abundant in Europe. Being a highly adaptive species, they have also adjusted to suburban and urban environments (Roca et al. 2014). In the current report, the first cardiovascular dirofilariosis cases in two European badgers in Greece are described.

## Materials and methods

In March 2023, in Heraklion, Crete Island (35.341846, 25.148254), an adult male European badger was found in an impaired physical condition, rescued, and admitted to “ANIMA” in Athens. From the detailed clinical examination upon admission, severe dehydration, pale mucous membranes, severe inspiratory dyspnea, and hyperthermia (39.6 °C) were noticed. Also, abnormal lung sounds (rhonchi) were heard on auscultation.

A 5-mL blood sample was drawn from the jugular vein and divided into EDTA, heparine lithium, and RIA tubes. Subsequently, the tubes were transported to a specialised and certified veterinary diagnostic laboratory (VET IN PROGRESS PLUS, Athens, Greece) for further haematological, biochemical, and immunological analyses for different pathogens, including *D. immitis*. More specifically, the highly sensitive and specific enzyme-linked immunosorbent assay (ELISA) “DiroCHEK ELISA Canine Heartworm Antigen Test” was used for the detection of the female adult *D. immitis* antigen, as well as a modified Knott’s test for the detection of microfilariae in the peripheral blood.

Supportive treatment immediately initiated, including fluid therapy i.v. with sodium chloride 0.9% (sodium chloride 0.9%, DEMO ΑΒΕΕ, Greece) at a dose rate of 60 ml/kg/day and supplemental oxygen flow by (800 ml/min). In addition, NSAIDs (meloxicam at a dose rate of 0.2 mg/kg/24 h SC -Metacam 5 mg/ml, Boehringer Ingelheim, Germany) and antibiotics (enrofloxacin at a dose rate of 5 mg/kg IM, -Baytril 5%, Bayer, Germany) were administered. Even though the badger was placed in the intensive care unit, it died a few hours after admission. In order to determine the potential cause of death, a thorough necropsy was carried out, during which all the organs of the thoracic and the abdominal cavity were examined via visual inspection, palpation, and incision of their parenchyma.

In July of the same year, a female European badger hit by a car in Chania, Crete Island (35.513828, 24.018038) was also admitted to “ANIMA” in Athens. The animal presented severe neurological clinical signs (lack of consciousness, blindness, nystagmus, rotational movements) due to craniocerebral trauma. Meloxicam was administered subcutaneously at a dose rate of 0.2 mg/kg (metacam 5 mg/ml, Boehringer Ingelheim, Germany) along with fluid therapy (sodium chloride 0.9%, DEMO ΑΒΕΕ, Greece) (90 ml/kg/day) and mannitol at a dose of 1.5 g/kg IV over 20 min (mannitol 20%, DEMO ΑΒΕΕ, Greece) by the veterinarian to try and stabilise the patient.

A 5-mL blood sample was drawn from the cephalic vein, which was then divided into EDTA, heparine lithium, and RIA tubes. The samples were sent to the same veterinary diagnostic laboratory (VET IN PROGRESS PLUS, Athens, Greece) for further haematological, biochemical, and immunological analyses for different pathogens, including *D. immitis* (“DiroCHEK Canine Heartworm Antigen Test Kit” and modified Knott’s test).

The animal died 3 h after its admission, and a detailed necropsy was performed.

## Results

For the first badger, haematology and biochemistry were unremarkable, with all values within reference intervals for the specific species. Furthermore, the animal was negative for *Leishmania* spp. (IFA—IgG),* Ehrlichia canis* (IFA—IgG & IgM), *Leptospira canicola* (IFA—IgG), *Leptospira icterohaemorrhagiae* (IFA—IgG), and Distemper virus (IFA – IgG & IgM). However, the badger tested positive for the heartworm antigen in the ELISA test and also for microfilariae in Knott’s test after examination under an optical microscope (Olympus, CX21 Microscope). The microfilariae were identified as *D. immitis* based on morphological keys for the specific species (Magnis et al. 2013).

Regarding the postmortem findings, no external injuries were detected during the autopsy, and the carcass had an emaciated body condition score (BCS). No secretions were noticed in the badger’s oral cavity and nostrils, and there were no abnormal findings in the subcutaneous tissue. All the abdominal cavity’s organs were normal in colour, texture, and size. Inside the thoracic cavity, the lungs had a firm and liver-like texture with a cranioventral distribution of the lesions, accompanied by interlobular emphysema.

**Fig. 1:**
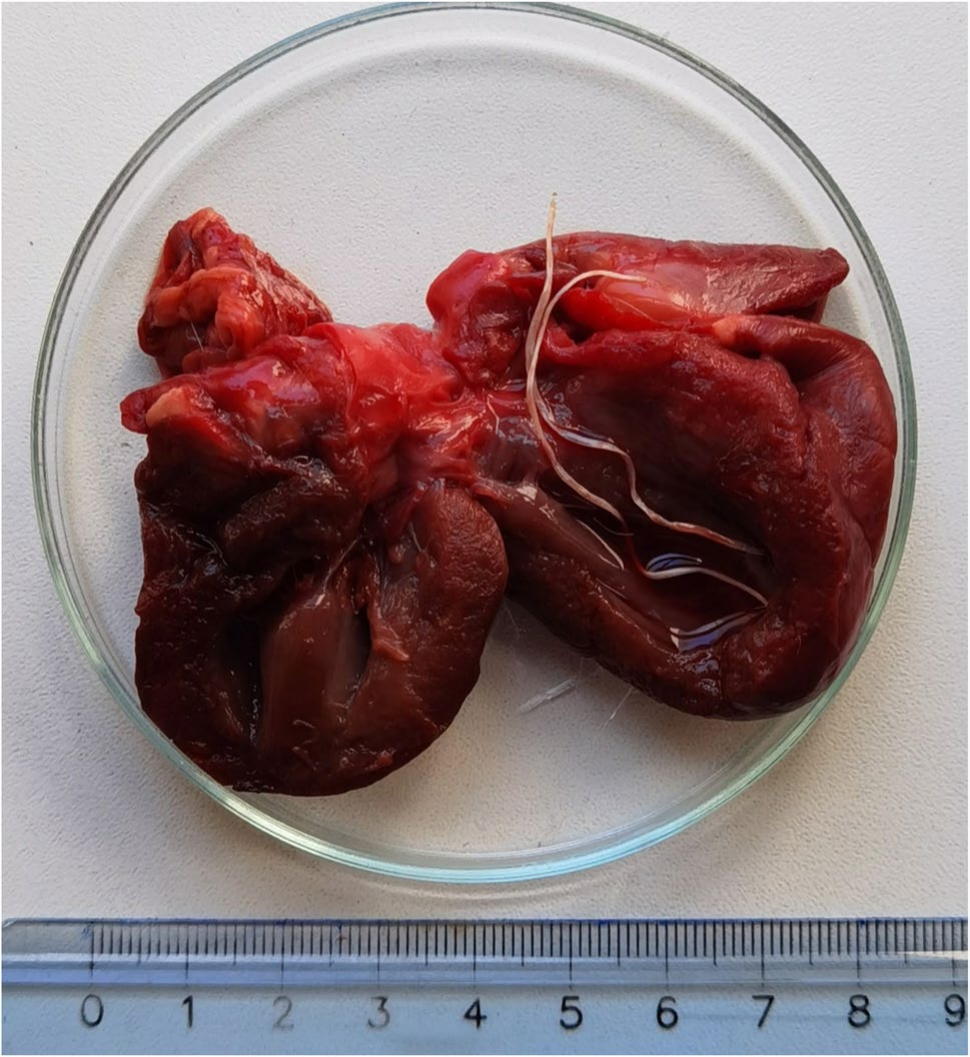
*Dirofilaria immitis* adults in the right ventricle of the heart of the first badger

Based on all the available diagnostic information, the first badger’s death was related to acute bronchopneumonia. Consequently, the heart, vena cava, proximal end of the pulmonary artery, and lungs were excised and visually examined for the presence of adult worms. Three nematodes were recovered from the heart’s right ventricle (Fig. 1) that were morphologically identified as *D. immitis* adults under a stereomicroscope (Olympus, Research Stereomicroscope System SZH10) using morphological keys for the specific species (Furtado et al. 2010). Their sex was determined based on tail morphology, resulting in one male and two female adults.

For the second badger, haematology and biochemistry were also unremarkable, with all values within reference intervals for the specific species. Lab results came back negative for *Leishmania* spp. (IFA—IgG), *Ehrlichia canis* (IFA—IgG & IgM), *Leptospira canicola* (IFA—IgG), and *Leptospira icterohaemorrhagiae* (IFA IgG). In contrast, the second badger tested positive for Distemper virus (IFA – IgG), for the heartworm antigen in the ELISA test, and also for *D. immitis* microfilariae in Knott’s test after examination under an optical microscope (Olympus, CX21 Microscope) according to the same published keys (Magnis et al. 2013).

Regarding the postmortem findings, external injuries were detected on its skull during the autopsy, and the carcass had a good BCS. No secretions were observed in the badger’s oral cavity and nostrils had and there were no abnormal findings in the subcutaneous tissue. All the abdominal and thoracic cavities’ organs were normal in colour, texture, and size. The heart, vena cava, proximal end of the pulmonary artery, and lungs were excised and visually examined for any adult worms. Three nematodes were recovered from the heart’s right ventricle that were morphologically identified as *D. immitis* adults under a stereomicroscope (Olympus, Research Stereomicroscope System SZH10) using the same morphological keys (Furtado et al. 2010). The adult worms’ sex was determined based on tail morphology, resulting in two males and one female adult. The measurements (length and width) of the three adults of *D. immitis* from the first badger and the three adults from the second badger are summarised in Table 1.

**Table 1:** Length, width, and sex of all the adult *D. immitis* nematodes isolated from the first and second badger after necropsy

*Dirofilaria immitis* adults	Badger isolated from	Sex	Length (cm)	Width (mm)
Adult 1	1	Male	10.8	1
Adult 2	1	Female	23.4	1
Adult 3	1	Female	22.7	1
Adult 4	2	Male	12.5	1
Adult 5	2	Male	12.1	1
Adult 6	2	Female	24.2	1

## Discussion

The current cases represent the second confirmed report of *D. immitis* infection in badgers (via antigen testing, Knott’s test, and necropsy), further supporting the theory that the badger constitutes an additional reservoir host for *D. immitis* in nature. Although the extent and duration of microfilaraemia remain unknown and require further investigation, the life cycle could be completed since a mosquito could acquire the microfilariae from the badger during a blood meal. The first *D. immitis* infection in a European badger was recorded in Romania (Ionică et al. 2022) and was the only badger infected out of the 237 examined. Future epidemiological studies should also focus on the molecular identification of this parasite in wildlife animals and investigating the presence of different haplotypes.

The present study’s findings also contribute to the epidemiological map of heartworm disease. In Greece, dirofilariosis is enzootic and widespread, particularly in the central and northern regions of the country (Polizopoulou et al. 2000). Moreover, most cases concern companion animals and there is another report of wildlife infection, in a brown bear (*Ursus arctos* Linnaeus, 1758) (Papadopoulos et al. 2017), recorded in northern Greece. Therefore, it is remarkable that these European badgers were infected in isolated areas (island) that are not considered endemic for dirofilariosis and where autochthonous cases are scarce.

Other wild animals, including golden jackals, brown bears, red foxes, and grey wolves, which serve as competent hosts for the parasite (Moroni et al. 2020), have never been detected in Crete. However, the European badgers of Crete are sympatric with a subspecies of European wildcat as well as with numerous hunting dogs, which are commonly infected with *D. immitis* (Papazahariadou et al. 1994). Although Crete Island currently seems unaffected by heartworm disease, its climatic conditions and the wide variety of Culicidae mosquitoes (Fotakis et al. 2022) are ideal for *D. immitis* to complete its life cycle. Therefore, the first case of cardiovascular dirofilariosis in wild animals on the island warrants further investigation into the local wildlife and canine population.

In regards to prevalence in wild carnivores, one study found that golden jackals present the highest prevalence in the Canidae family, followed by red foxes. It is still unclear if red foxes serve as reservoir hosts for this parasite, as patent infections are uncommon (Hubert et al. 1980; Ionică et al. 2022). Regarding mustelids, *D. immitis* infections have been identified in ferrets (*Mustela putorius* Linnaeus, 1758) (Campbell and Blair 1978) and Eurasian otters (*Lutra lutra* Linnaeus, 1758) (Ionicǎ et al. 2017).

As the spectrum of *D. immitis* suitable hosts increases, veterinarians should be alert and raise awareness among pet owners regarding prevention measures for their companion animals such as dogs and cats. Additionally, given the zoonotic potential of the parasite, humans living in Crete Island should also be aware of the disease.

## Conclusions

In their search for food, wild animals have become more common in urban areas and have little or no fear of humans, as never before. Consequently, the transmission risk of pathogens, such as parasites, among humans and wild and domestic animals has increased. Regarding dirofilariosis, wild animals are excellent sentinels for assessing human infection risk. Future longitudinal studies should investigate the prevalence of *D. immitis* in wild animals, aiming to determine new host-parasite associations (Haydon et al. 2002) and thus clarify the role of urban wild animal populations in the epidemiology of heartworm disease.

## Data Availability

Data supporting the reported results are included in the article.

## References

[CR1] Campbell WC, Blair LS (1978). *Dirofilaria immitis*: experimental infections in the ferret (*Mustela putorius furo*). J Parasitol.

[CR2] Diakou A, Kapantaidakis E, Tamvakis A, Giannakis V, Strus N (2016). *Dirofilaria* infections in dogs in different areas of Greece. Parasit Vectors.

[CR3] Fotakis EA, Mavridis K, Kampouraki A (2022). Mosquito population structure, pathogen surveillance and insecticide resistance monitoring in urban regions of Crete, Greece. PLoS Negl Trop Dis.

[CR4] Furtado AP, Melo FTV, Giese EG, Dos Santos JN (2010). Morphological redescription of *Dirofilaria immitis*. J Parasitol.

[CR5] Genchi C, Rinaldi L, Mortarino M, Genchi M, Cringoli G (2009). Climate and *Dirofilaria* infection in Europe. Vet Parasitol.

[CR6] Gortazar C, Castillo JA, Lucientes J, Blanco JC, Arriolabengoa A, Calvete C (1994). Factors affecting *Dirofilaria immitis* prevalence in red foxes in northeastern Spain. J Wildl Dis.

[CR7] Haydon DT, Cleaveland S, Taylor LH, Laurenson MK (2002). Identifying reservoirs of infection: a conceptual and practical challenge. Emerg Infect Dis.

[CR8] Hoch H, Strickland K (2008). Canine and feline dirofilariasis: life cycle, pathophysiology, and diagnosis. Compend Contin Educ Vet.

[CR9] Hubert GF, Kick TJ, Andrews RD (1980). *Dirofilaria immitis* in red foxes in Illinois. J Wildl Dis.

[CR10] Ionică AM, Deak G, Boncea R, Gherman CM, Mihalca AD (2022) The European badger as a new host for *Dirofilaria immitis* and an update on the distribution of the heartworm in wild carnivores from Romania. Pathogens 11:. 10.3390/pathogens11040420 10.3390/pathogens11040420 PMC9032528 35456095

[CR11] Ionicǎ AM, Matei IA, D’Amico G (2017). Filarioid infections in wild carnivores: a multispecies survey in Romania. Parasit Vectors.

[CR12] Magnis J, Lorentz S, Guardone L, et al (2013) Morphometric analyses of canine blood microfilariae isolated by the Knott’s test enables *Dirofilaria immitis* and *D. repens* species-specific and *Acanthocheilonema* (syn. *Dipetalonema*) genus-specific diagnosis. Parasites and Vectors 6:1. 10.1186/1756-3305-6-48 10.1186/1756-3305-6-48 PMC3598535 23442771

[CR13] Moroni B, Rossi L, Meneguz PG (2020). *Dirofilaria immitis* in wolves recolonizing northern Italy: are wolves competent hosts?. Parasit Vectors.

[CR14] Otranto D, Dantas-Torres F, Brianti E (2013). Vector-borne helminths of dogs and humans in Europe. Parasit Vectors.

[CR15] Otranto D, Deplazes P (2019). Zoonotic nematodes of wild carnivores. Int J Parasitol Parasites Wildl.

[CR16] Papadopoulos E, Komnenou A, Poutachides T (2017). Detection of *Dirofilaria immitis* in a brown bear (*Ursus arctos*) in Greece. Helminthol.

[CR17] Papazahariadou MG, Koutinas AF, Rallis TS, Haralabidis ST (1994). Prevalence of microfilaraemia in episodic weakness and clinically normal dogs belonging to hunting breeds. J Helminthol.

[CR18] Polizopoulou ZS, Koutinas AF, Saridomichelakis MN (2000). Clinical and laboratory observations in 91 dogs infected with *Dirofilaria immitis* in northern Greece. Vet Rec.

[CR19] Roca CP, La Haye MJJ, Jongejans E (2014). Environmental drivers of the distribution and density of the European badger (*Meles meles*): a review. Lutra.

[CR20] Rossi L, Pollono F, Meneguz PG, Gribaudo L, Balbo T (1996). An epidemiological study of canine filarioses in North-West Italy: what has changed in 25 years?. Vet Res Commun.

[CR21] Simón F, Siles-Lucas M, Morchón R (2012). Human and animal dirofilariasis: the emergence of a zoonotic mosaic. Clin Microbiol Rev.

[CR22] Tatem AJ, Rogers DJ, Hay SI (2006). Global transport networks and infectious disease spread. Adv Parasitol.

[CR23] Tolnai Z, Széll Z, Sproch Á, Szeredi L, Sréter T (2014). *Dirofilaria immitis*: an emerging parasite in dogs, red foxes and golden jackals in hungary. Vet Parasitol.

[CR24] Wixsom MJ, Green SP, Corwin RM, Fritzell EK (1991). *Dirofilaria immitis* in coyotes and foxes in Missouri. J Wildl Dis.

